# Efficacy of a Selective Binder of α_V_β_3_ Integrin Linked to the Tyrosine Kinase Inhibitor Sunitinib in Ovarian Carcinoma Preclinical Models

**DOI:** 10.3390/cancers11040531

**Published:** 2019-04-13

**Authors:** Andrea Sartori, Cristina Corno, Michelandrea De Cesare, Eugenio Scanziani, Lucia Minoli, Lucia Battistini, Franca Zanardi, Paola Perego

**Affiliations:** 1 Food and Drug Department, University of Parma, Parco Area delle Scienze 27A, 43124 Parma, Italy; andrea.sartori@unipr.it (A.S.); lucia.battistini@unipr.it (L.B.); 2 Molecular Pharmacology Unit, Fondazione IRCCS Istituto Nazionale dei Tumori, Via Amadeo 42, 20133 Milan, Italy; cristina.corno@istitutotumori.mi.it (C.C.); laura.zanesi@istitutotumori.mi.it (M.D.C.); paola.perego@istitutotumori.mi.it (P.P.); 3 Department of Veterinary Medicine, University of Milan, Via Celoria 10, 20133 Milan, Italy; eugenio.scanziani@unimi.it (E.S.); lucia.minoli@unimi.it (L.M.); 4 Mouse and Animal Pathology Lab, Fondazione Filarete, Viale Ortles 22/24, 20139 Milan, Italy

**Keywords:** integrins, molecular conjugates, ovarian carcinoma, RGD ligands, RTK inhibitors, sunitinib

## Abstract

Ovarian carcinoma, the most lethal gynecological cancer, is characterized by late diagnosis, with drug resistance limiting the efficacy of platinum-based therapy. Since some integrins are upregulated in cancer, including ovarian carcinoma, they represent a potential target for drug delivery. Receptor tyrosine kinases are also deregulated in cancer and their expression has been associated with drug resistance. Here, the antitumor effects of three conjugates possessing a selective binder of the extracellular portion of integrin α_V_β_3_ covalently linked to the tyrosine kinase inhibitor sunitinib were investigated in cisplatin-sensitive and -resistant ovarian carcinoma cells expressing both tyrosine kinase VEGFR2 and α_V_β_3_ at different levels. We found that one of the three compounds was active in inhibiting the growth of both drug-sensitive and -resistant cells in the micromolar range with a slightly increased potency in resistant cells as compared to sunitinib. The same compound markedly impaired cell migratory and invasive abilities and reduced paxillin phosphorylation. Antitumor activity studies in IGROV-1/Pt1 cells xenografted in nude mice revealed a striking activity of this conjugate versus sunitinib. Taken together, our results support the interest of integrin-targeted sunitinib conjugates for the treatment of drug-resistant tumors.

## 1. Introduction

Ovarian carcinoma is the most lethal gynecological cancer as it is often diagnosed at an advanced stage [1]. Early detection increases the probability of a favorable prognosis, but it is not frequent due to lack of early disease symptoms. Late diagnosis makes the disease difficult to cure in spite of the initial efficacy of platinum-based treatment [2,3]. In fact, a persistent response to treatment is not achieved and drug resistance is developed [4,5]. The pathways sustaining drug resistance of ovarian carcinoma are multiple and involve, among the diverse factors, drug accumulation impairment, loss of DNA mismatch repair, increased nucleotide excision repair, and hyperactivation of receptor tyrosine kinases (RTKs) [5,6,7]. Such receptors, frequently deregulated in cancer, are implicated in the regulation of multiple processes including cell survival and angiogenesis [8]. The latter phenomenon is mainly sustained by vascular endothelial growth factor receptor (VEGFR) activation, as shown by several reports in different cancer types [9]. Targeting of angiogenesis is already pursued in the clinics in ovarian cancer, as shown by the approval of the anti-VEGF antibody Bevacizumab as frontline treatment after surgery. Although anti-angiogenesis therapy in ovarian cancer has been shown to prolong disease-free survival [10], there is an urgent need to identify novel anti-angiogenic drugs with minimal side-effects. In this context, an innovative approach is represented by the use of tumor-targeting strategies aimed at delivering both conventional and targeted drugs selectively to tumor cells [11,12]. This approach has already generated promising preclinical results translatable to the clinical setting [13,14,15].

The use of selective binders of the extracellular portion of integrin α_V_β_3_ covalently linked to targeted agents may result in the selective inhibition of tumor cell growth and, given the pattern of overexpression of α_V_β_3_ also present in endothelial cells, these conjugates represent an opportunity for a dual targeting strategy impairing the microenvironment ability to sustain tumor cell growth.

**Figure 1:**
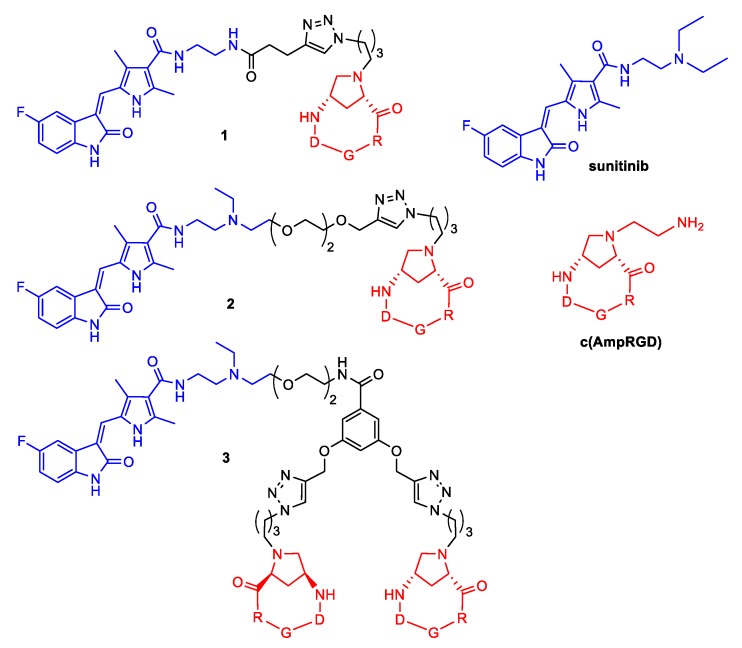
Molecular structures of conjugates **1**–**3** and the parent sunitinib and c(AmpRGD) components.

Recently, a new class of molecular conjugates was launched, where the α_V_β_3_ integrin binder c(AmpRGD) [16,17] (alias aminoproline-based RGD cyclotetrapeptide) was covalently connected to the anti-tumor and anti-angiogenic RTK inhibitor sunitinib (compounds **1**–**3**, Figure 1) [18]. These compounds proved chemically robust, competent, and selective binders of α_V_β_3_-overexpressing endothelial progenitor cells and melanoma cells, capable of (at least partially) entering these cells and inhibiting their proliferation. Furthermore, they demonstrated an ability to impair tumor-associated angiogenesis in vitro and in vivo, and to significantly decrease xenografted melanoma tumor growth in mice [18,19].

Based on this background, the present study was designed to selectively target ovarian carcinoma cells characterized by aggressive features in terms of response to treatment (i.e., drug resistance) and pro-invasive behavior. We elected to use conjugates **1**–**3** in this endeavor considering that (i) the integrin-recognizing moiety could ensure cell-selective accumulation on α_V_β_3_-overexpressing ovarian carcinoma cells and possibly provide internalization via integrin-mediated endocytosis, (ii) once entered the cells, the sunitinib moiety could be available to exert its intracellular inhibitory effect, and (iii) the overall conjugates could alter the α_V_β_3_-VEGFR2 crosstalk thus impacting the downstream intracellular pathways related to these receptors [18,19].

We demonstrated that conjugates **1**–**3** inhibited the growth and migration ability of cisplatin-sensitive ovarian carcinoma cells at low micromolar concentrations. Furthermore, conjugate **3** was able to inhibit cell growth and markedly reduce the migration potential of cisplatin-resistant ovarian carcinoma cells, even better than sunitinib. Compounds **1**–**3** reduced the phosphorylation of paxillin, a focal adhesion-associated, phosphotyrosine-containing protein implicated in VEGF signaling [20].

We also showed that conjugate **3** significantly decreased the growth of cisplatin-resistant carcinoma cells implanted in nude mice as compared to sunitinib even at lowered dosage, supporting the notion that these integrin-targeted sunitinib conjugates can be promising tools against drug-resistant tumors.

## 2. Results

### 2.1. Synthesis of Conjugates ***1**–**3***

The molecular conjugates **1**–**3** and the unconjugated reference compound c(AmpRGD) (Figure 1) were prepared according to reported procedures [17,18].

### 2.2. Levels of VEGFR2

**Figure 2:**
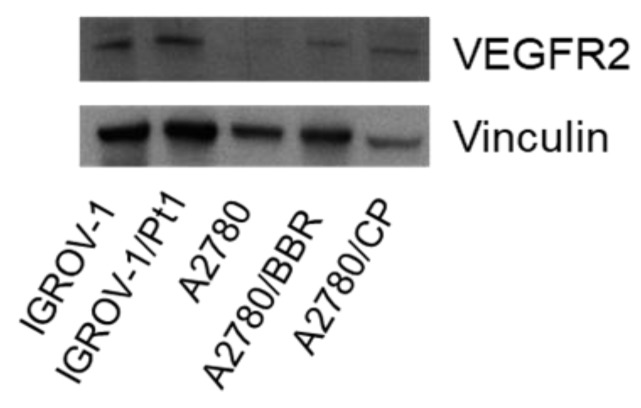
Western blot analysis of VEGFR2 levels in different ovarian carcinoma cell lines. Western blot analysis was carried out in ovarian carcinoma exponentially growing cells. Control loading is shown by vinculin. One experiment representative of three is reported. For whole Western blot analysis of VEGFR2 levels and band intensities quantification of VEGFR2 levels in different ovarian carcinoma cell lines, see Figure S3 and Table S1, respectively.

Deregulated expression of receptor tyrosine kinases (RTKs) may have a prognostic relevance in ovarian carcinoma and activation of survival pathways downstream of RTKs is known to be involved in cellular drug resistance both to conventional drugs and to targeted agents [5,6]. Thus, we examined the expression of VEGFR2 in cisplatin-sensitive and -resistant cells by western blot analysis. We found that VEGFR2 was expressed in various ovarian carcinoma cell lines including IGROV-1, IGROV-1/Pt1, and the two drug-resistant variants of A2780 cells (A2780/BBR and A2780/CP) (Figure 2).

### 2.3. Cell Sensitivity of Cisplatin-Sensitive and -Resistant IGROV-1 Ovarian Carcinoma Cell Lines

The three conjugates **1**–**3**, as well as their parent compound c(AmpRGD), are characterized by selective binding capability toward the α_V_β_3_ integrin receptor, as previously demonstrated in both cell-free and cellular assays [17,18,19]. Specifically, the binding competence of these compounds toward the isolated α_V_β_3_ receptor was proven, with IC_50_ values in the competitive displacement of biotinylated vitronectin, a natural ligand of the α_V_β_3_ receptor, being comprised in the low nanomolar range (compound **1**, IC_50_ α_V_β_3_ 1.24 nM, compound **2**, IC_50_ α_V_β_3_ 5.1 nM, compound **3**, IC_50_ α_V_β_3_ 3.8 nM, c(AmpRGD), IC_50_ α_V_β_3_ 6.1 nM) [17,18]. Thus, it was demonstrated that the presence of both the sunitinib moiety and the linker did not compromise the good α_V_β_3_-integrin targeting capability of these covalent conjugates [18]. These compounds were also assayed for their ability to inhibit the adhesion to vitronectin of both α_V_β_3_-overexpressing and α_V_β_3_-lacking cells (namely, α_V_β_3_-positive endothelial progenitor cells EPCs and melanoma cells M21, as well as α_V_β_3_-lacking human prostate carcinoma cells PC3 and human erythroleukemia cells K562); the results showed that conjugates **1**–**3** inhibited cell adhesion to vitronectin of α_V_β_3_-overexpressing cells in the low micromolar range, while the c(AmpRGD) parent compound did not impact at all on cell adhesion to vitronectin in α_V_β_3_-lacking cells [18,19].

**Figure 3:**
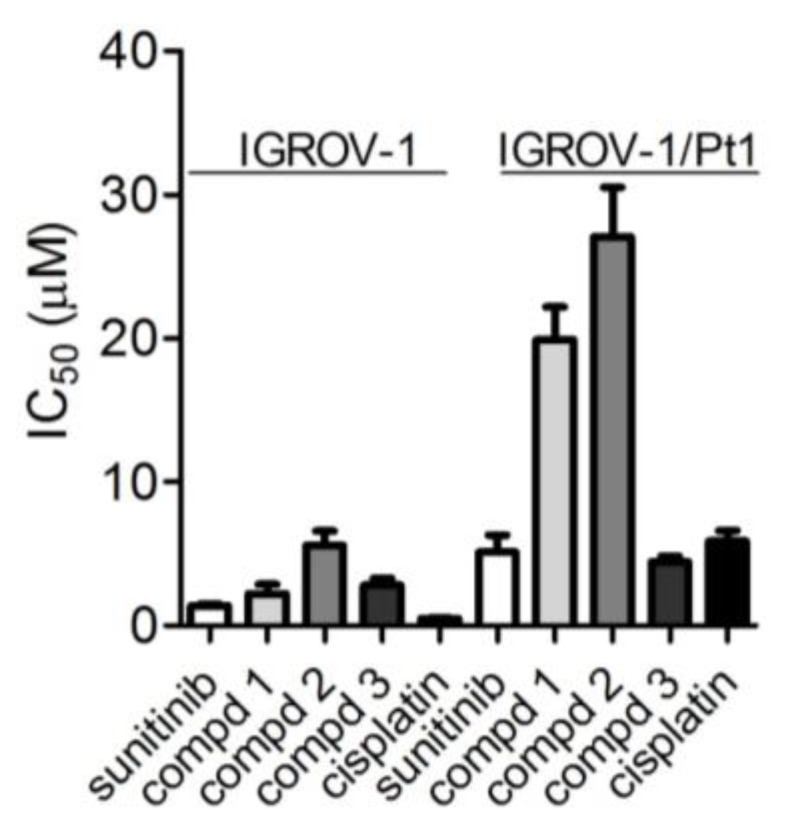
Sensitivity to sunitinib, conjugates **1**–**3** and cisplatin as assessed by cell growth inhibition assays. Cells were seeded and 24 h later exposed to the compounds for 72 h. Cells were then counted using a cell counter. IC_50_ is defined as the concentration inhibiting cell growth by 50%. The IC_50_ values of the c(AmpRGD) per se were 6.04 ± 2.8 μM in IGROV-1 cells and >30 μM in IGROV-1/Pt1 cells. Sensitivity relative to sunitinib is: 1.59, 4.00, 2.01 for compounds **1**, **2**, and **3**, respectively, in IGROV-1 cells, and 3.88, 5.28, 0.86 for compounds **1**, **2**, and **3**, respectively, in IGROV-1/Pt1 cells.

Based on these precedents, and considering that (i) both cisplatin-sensitive IGROV-1 cell line and its cisplatin-resistant IGROV-1/Pt1 counterpart express VEGFR2 (vide supra); (ii) both IGROV-1 and IGROV-1/Pt1 cells express the α_V_β_3_ integrin, with the latter expressing higher levels of the α_V_β_3_ integrin as compared to parental cells (Figure S1) [13]; and (iii) IGROV-1/Pt1 cells exhibit a stable drug-resistant phenotype with a degree of cisplatin resistance of 11.5, the sensitivity of these IGROV-1 and IGROV-1/Pt1 cells to the three conjugates **1**–**3** was assessed by growth inhibition assays after 72 h drug exposure (Figure 3).

The three conjugates exhibited a marked capability to inhibit cell growth in the parental cell line, the IC_50_ values being in the low micromolar range in the IGROV-1 cell line, whereas a reduced inhibitory effect was observed for compounds **1** and **2** in IGROV-1/Pt1 cells (for **1**, *p* = 0.0017; for **2**, *p* = 0.0039 by two-tail Student’s *t*-test, *n* = 3). In fact, only compound **3** was active in the low micromolar range in the cisplatin-resistant cell line and, differently from the other conjugates, this compound tended to display a marginally reduced sensitivity as compared to IGROV-1 cells (*p* = 0.0542, by two-tail Student’s *t*-test, *n* = 3). Cell sensitivity to the parent unconjugated c(AmpRGD) ligand and to sunitinib was also examined for comparison. The c(AmpRGD) ligand displayed an inhibitory effect in the parental cell line but not in the resistant variant. The growth inhibitory activity of sunitinib was decreased in the platinum-resistant cells (*p* = 0.0086, by two-tail Student’s *t*-test, *n* = 4) to a larger extent than conjugate **3**, whose activity was comparable to that of sunitinib. In parental cells, on the other hand, a slightly reduced effect was observed for conjugate **3** as compared to sunitinib, (*p* = 0.0106 by Student’s *t*-test, *n* = 3).

### 2.4. Modulation of Cell Migratory and Invasive Abilities and Modulation of Downstream Targets of VEGFR2 of Cisplatin-Sensitive and -Resistant IGROV-1 Ovarian Carcinoma Cell Lines

**Figure 4:**
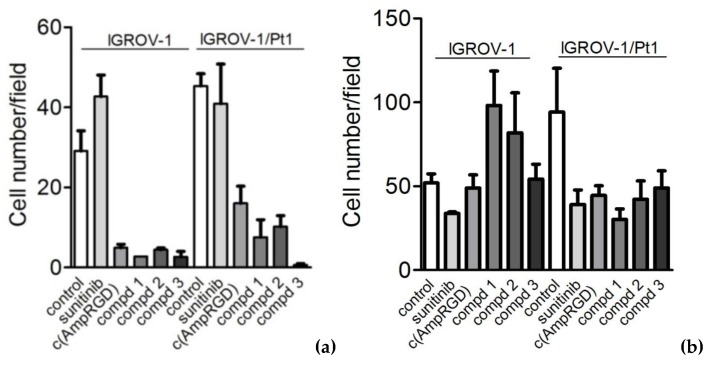
Modulation of migratory and invasive abilities of IGROV-1 and IGROV-1/Pt1 cells by sunitinib and selective binders of α_V_β_3_ integrin namely, c(AmpRGD) alone or linked to sunitinib (compounds **1**–**3**). Cells were subjected to migration (**a**) and invasion assays (**b**) in serum-free medium using transwell plates after exposure to the compounds (10 μM). Migrating and invading cells were counted under a light microscope. Columns represent cell numbers/field (± SE; *n* = 3).

Because VEGFR2 is implicated in sustaining tumor aggressiveness, in particular cell migration and invasive abilities [20,21], we examined the effects of all conjugates on these aggressive cell features (Figure 4). IGROV-1/Pt1 cells displayed a significantly higher migratory and invasive potential as compared to parental cells (*p* < 0.05, unpaired Student’s *t*-test). Cells were exposed for 24 h to concentrations of agents (10 μM) unable to affect viability at that time and—using transwell assays—we found that all (and the sole) RGD-containing molecules were able to inhibit cell migration of both cell lines and, in particular, compound **3** produced the most striking effect in almost completely inhibiting migration of resistant cells (Figure 4a). As for the modulation of invasiveness (Figure 4b), while IGROV-1 cells were not inhibited at all by RGD-containing molecules, cisplatin-resistant cells resulted inhibited by both sunitinib and RGD-compounds to almost the same extent.

**Figure 5:**
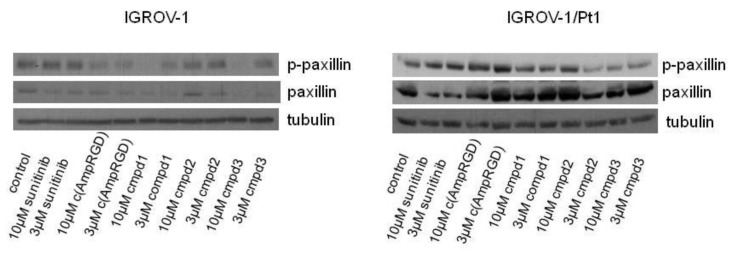
Western blot analysis of paxillin and phosphorylated-paxillin (p-paxillin) levels in IGROV-1 and IGROV-1/Pt1 cells. Cells were exposed for 24 h to sunitinib or to selective binders of α_V_β_3_ integrin linked to sunitinib (compounds **1**–**3**) at the indicated concentrations and then harvested for western blot analysis. Tubulin was used as loading control. A representative experiment is shown (for quantification of band intensities, see Table S2). For whole Western blot analysis of paxillin and phospho-paxillin levels in IGROV-1 and IGROV-1/Pt1 cells, see Figure S4.

An analysis of cell response to treatment was undertaken by western blot analysis, by evaluating the inhibition of phosphorylation of paxillin, a phosphotyrosine-containing protein downstream the VEGF/α_V_β_3_ receptor signaling. As shown in Figure 5 and Table S2, we found that in cisplatin-sensitive cells, all RGD compounds reduced the phosphorylation of paxillin at 10 μM concentration, while the treatment of the cisplatin-resistant cells with RGD-sunitinib conjugates **1**–**3** decreased the levels of phosphorylated paxillin at both 10 μM and 3 μM concentrations.

### 2.5. Cell Sensitivity and Modulation of Cell Migratory and Invasive Abilities of Cisplatin-Sensitive A2780 Ovarian Carcinoma Cell Line

In the perspective of developing therapeutic approaches precisely tailored to tumor cell alterations, we extended the analysis of selected effects of the conjugates under investigation to the A2780 cell line which displays still detectable levels of VEGFR2, but lower than those of IGROV-1/Pt1 cells (Figure 2). These cells also express reduced levels of the α_V_β_3_ integrin as compared to IGROV-1/Pt1 cells, as shown by flow cytometry analysis (Figure S1). Thus, the sensitivity of the A2780 ovarian carcinoma cell line to sunitinib and the three conjugates **1**–**3** was firstly assessed by growth inhibition assays after 72 h drug exposure (Table S3). All the tested compounds were active in the low micromolar range, with sunitinib-linked conjugates **1** and **3** behaving similarly to sunitinib. In addition, A2780 cells were exposed for 24 h to concentrations of agents (0.3 μM) unable to affect viability and, using transwell assays, we found that the sunitinib-containing molecules, both the free drug and RGD-conjugates **1**–**3**, were able to inhibit cell migration and invasion, while the sole RGD molecule showed pro-invasive and pro-migratory behavior (Figure S2).

### 2.6. In Vivo Antitumor Activity

**Figure 6:**
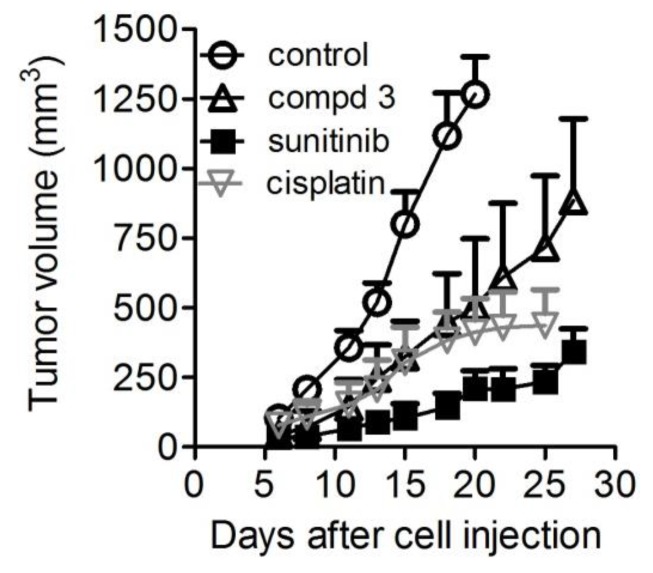
Antitumor activity of conjugate **3** in IGROV-1/Pt1 tumors. The effects of i.p. compound **3**, 20 mg/kg (having a sunitinib-equivalent loading 9 times lower than parental sunitinib), oral sunitinib, 40 mg/kg, qd×5/w×4w and intravenous cisplatin (4.5 mg/Kg, q7d×3) were evaluated on the growth of the IGROV-1/Pt1 carcinoma cells s.c. injected into the right flank of female nude mice (10^7^ cells/mouse) on day 0. Treatment started on day 4: compound **3** (open up triangle), sunitinib (filled square), cisplatin (down triangle) or control (open circle). Experimental groups consisted of seven animals. Growth curves of the tumors are shown. Points report mean values of tumor volumes. Bars: SD.

Given the promising results displayed by conjugate **3** in vitro, the antitumor activity of compound **3** as compared to sunitinib and cisplatin was assayed in nude mice bearing s.c. the IGROV-1/Pt1 human ovarian carcinoma. The results are shown in Table 1 and Figure 6. Sunitinib could highly impair tumor growth of IGROV-1/Pt1 xenografts with a TVI value of 84% (*p* < 0.0001, 20 days after cell injection, Student’s *t*-test of control versus sunitinib, *n* = 7). Interestingly, even though endowed with a load of sunitinib about nine times lower, compound **3** was still able to induce a TVI of 60% (*p* < 0.0001, 20 days after cell injection, Student’s *t*-test of control versus compound **3**, *n* = 7). Treatment was well tolerated. No toxic death occurred in treated mice. Cisplatin administered according to its clinical route produced a TVI of 67% (*p* < 0.0001, 20 days after cell injection, Student’s *t*-test of control versus cisplatin, *n* = 7).

**Figure 7:**
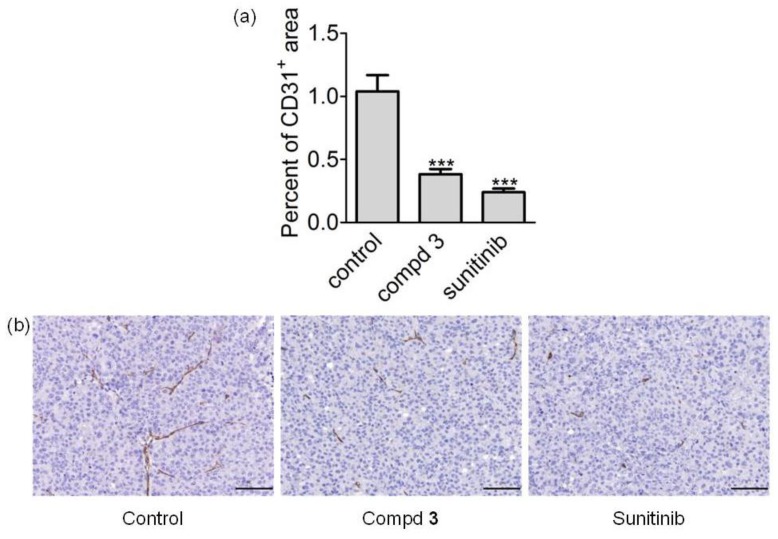
Evaluation of microvessels after treatment. Histopathological analysis of IGROV-1/Pt1 xenograft samples after treatment with compound **3** or sunitinib. (**a**) Quantitative analysis of tumor associated vasculature. CD31-positive area was measured in three randomly selected 200× microscopic fields from seven samples per group. The reported numbers correspond to the mean of CD31 positive areas in analyzed groups. Tumors were obtained from mice sacrificed 24 h after the last treatment; *** correspond to *p* < 0.001 by One Way ANOVA followed by Bonferroni Multiple Comparison test. (**b**) Representative 200× microscopic fields from a control group sample, and tumor from a sample treated with compound **3** and sunitinib. The scale bar corresponds to 100 μm.

To investigate the vascular alterations induced by the treatment, we performed an immunohistochemistry analysis of microvessels evaluating CD31 positivity in tumor samples collected after treatment with compound **3**, sunitinib, and control mice. We observed that endothelial area (CD31 positive area) was significantly lower in tumor samples obtained from mice treated with conjugate **3** or sunitinib than control animals, indicating an anti-angiogenic effect of the two molecules (*p* < 0.05 by ANOVA followed by Bonferroni test) (Figure 7).

## 3. Discussion

Small molecule-drug conjugates represent a recent class of drugs with the potential to target tumor-specific or overexpressed antigens [11,12,22,23]. Since both RTK and some integrin subtypes such as α_V_β_3_ are overexpressed in cancer cells, agents directed to both these targets appear to be extremely promising, also taking into account the tumor targeting properties of integrin ligands [18,19,24]. In particular, α_V_β_3_ and VEGFR2, which are expressed in ovarian cancer, are implicated in signaling networks sustaining tumor cell survival and angiogenesis. Although ovarian carcinoma is not a frequent cancer, its outcome is poor because of its diagnosis as advanced disease and the development of drug resistance. Such resistance can be sustained by multiple mechanisms, including loss of DNA mismatch repair and enhanced nucleotide excision repair which in principle might also be sustained by the activation of survival pathways [25].

In the present study, we used different α_V_β_3_- and VEGFR2-positive ovarian carcinoma cell lines including drug-resistant variants to test the effects of conjugates **1**–**3** made by the covalent junction of a selective binder of the extracellular portion of integrin α_V_β_3_ to the RTK inhibitor sunitinib. The α_V_β_3_ binder was obtained from a series of aminoproline-based RGD cyclotetrapeptides of type c(AmpRGD) (Figure 1), as previously reported [16,17]. A selective and marked binding capability of both unconjugated and variously conjugated c(AmpRGD)-containing molecules toward the α_V_β_3_ integrin receptor has been previously reported by us in both cell-free and cell-based assays [14,17,18,19,26]. Furthermore, c(AmpRGD)-sunitinib conjugates **1**–**3** were previously proven to be partially internalized in both endothelial progenitor cells and M21 melanoma cells, and an active role of integrin α_V_β_3_ during cell uptake was demonstrated [18,19]. In addition, c(AmpRGD)-sunitinib conjugate **3** demonstrated good inhibitory activity against human recombinant VEGFR2 in the nanomolar range [18], demonstrating that the covalent conjugation of these two moieties could preserve the targeting abilities of the individual components.

In this study, we investigated the effects of conjugates **1**–**3** on both cisplatin-sensitive IGROV-1 and cisplatin-resistant IGROV-1/Pt1 cells that were found to express VEGFR2 (Figure 2). In addition, the drug-resistant cell line has been previously shown to display increased α_V_β_3_ levels as compared to the parental one [13] (Figure S1), and was therefore considered a unique model to investigate the efficacy of these dual compounds. The three conjugates displayed a marked antiproliferative activity in parental cells, with IC_50_ values in the low micromolar range, as shown by growth inhibition assays (Figure 3). The activity of compounds **1** and **2** was markedly reduced in cisplatin-resistant cells, while conjugate **3** maintained its antiproliferative activity even more than sunitinib.

An increased aggressiveness of IGROV-1/Pt1 cells in terms of migratory and invasive abilities has been linked to Axl overexpression [27]. Of note, here we observed that all conjugates **1**–**3** were capable of inhibiting cell migration in both IGROV-1 and IGROV-1/Pt1, with a striking effect of compound **3** on the migratory ability of resistant cells that was almost completely abolished (Figure 4). An inhibition of cell invasive ability was also observed. It has to be noted that the drug concentrations used for these experiments were equimolar (10 μM) and cells seeded for evaluation of migratory and invasive capability were viable.

Moreover, the A2780 cell line, exhibiting detectable levels of VEGFR2 and levels of α_V_β_3_ lower than IGROV-1/Pt1 cells, was also examined in cell migration and invasion assays, and we found that, again, conjugates **1**–**3** (at 0.3 μM concentrations) displayed the capability to inhibit cell aggressiveness, although to a reduced extent than in IGROV-1/Pt1 cells. Taken together, these results suggest that this class of compounds might be useful in ovarian carcinoma as novel therapeutic agents, but it appears to be important to consider the level of expression of VEGFR2 and α_V_β_3_ to target cells coexpressing both targets.

An interesting observation emerging from western blot analysis was the capability of the conjugated compounds **1**–**3** to reduce paxillin phosphorylation, evident in cisplatin-sensitive cells, and still achievable in the cisplatin-resistant cells at both 10 μM and 3 μM concentrations (Figure 5 and Table S2). Outside-in signaling via growth factor receptors and integrins has been shown to involve both non-RTKs such as c-Src, and RTKs, with triggering of signaling resulting in activation of PI3 kinase-Akt and canonical MAPK pathways [20]. Paxillin is a scaffold protein acting by recruiting signaling proteins to the plasma membrane and when it is phosphorylated by FAK or Src, additional binding sites for adaptor proteins are generated; the inhibition of its phosphorylation is therefore relevant to reduce the activation of oncogenic pathways with key roles in cancer cell proliferation and chemoresistance such as Akt and ERK1/2.

Given the interesting results obtained in these in vitro assays by conjugated compounds **1**–**3**, and in particular considering the good performance of compound **3** in inhibiting cell growth and preventing tumor cell aggressiveness in vitro, this last dual conjugate **3** was elected as the most promising candidate for advancing in vivo studies. The IGROV-1/Pt tumor model, in which cisplatin —usually displaying a marked activity in ovarian cancer xenografts—exhibited a significant but not striking activity due to the acquired resistant phenotype (Table 1 and Figure 6), was used for an evaluation of antitumor activity. In this regard, it has to be highlighted that compound **3** was used at 20 mg/Kg since this dose was previously shown to be well-tolerated by mice [18,19] and also to evaluate the impact of the in vivo treatment using a dosage expected to be devoid of toxicity. Sunitinib, instead, was tested at its optimal dose using the clinically relevant route, i.e., oral administration [28,29]. Under such conditions, the dose of the sunitinib moiety within conjugate **3** was around 9 (8.86) times lower than that delivered to mice treated with sunitinib malate (Figure 6). Thus, although the TVI observed for sunitinib-treated mice (84%) was greater than that of compound **3**-treated mice (60%), these results strongly support the tumor inhibitory potential of this conjugate. In keeping with the observed antitumor activity, when examining endothelial area (Figure 7), we observed a striking effect induced by compound **3**, as well as sunitinib, in impairing microvessel structure.

## 4. Materials and Methods

### 4.1. Drugs

Cisplatin (Teva Pharma Italia S.r.l., Nerviano, Italy) was diluted in saline. Sunitinib (as malate salt, >99% purity, Selleckchem, Munich, Germany) was dissolved and diluted in DMSO. Conjugates **1**–**3** and c(AmpRGD) (as TFA salts, 96–98% purity after semipreparative reverse-phase HPLC purification) were synthesized according to reported procedures [17,18]. For in vivo studies, compound **3** and cisplatin were dissolved in saline, and sunitinib was dissolved in DMSO and carboxymethylcellulose 0.5% (10% and 90%, respectively). Drugs were delivered in a volume of 10 mL/kg of body weight.

### 4.2. Cell Lines and Growth Conditions

The human ovarian carcinoma cell lines IGROV-1 (from Dr. Benard, Villjuif, France), A2780 (from Prof. Ozols, Bethesda, MD, USA), and the cisplatin-resistant variants—IGROV-1/Pt1, A2780/CP, and A2780/BBR—were obtained as previously described and were cultured in RPMI-1640 medium (Lonza, Basel, Switzerland), supplemented with 10% FBS (Gibco, Life Technologies, Carlsbad, CA, USA) [30,31]. Cells were thawed from frozen stocks and cultured for no more than 20 passages. Cell identity was authenticated through microsatellite analysis by the AmpFISTR Identifiler PCR Amplification Kit (Applied Biosystem, Carlsbad, CA, USA). Cell sensitivity to drugs was measured by growth inhibition assays based on cell counting. At 24 h after seeding, cells were exposed to the drugs for 72 h. Cells were counted with a cell counter at the end of drug incubation. IC_50_ is defined as the drug concentration producing 50% decrease of cell growth. Statistical analysis to compare IC_50_ values was carried out by two-side Student’s *t*-test using GraphPad Prism (version 5.02, GraphPad Software Inc., La Jolla, CA, USA).

### 4.3. Cell Migration and Invasion Assays

Cell migration and invasion assays were carried out as previously described [27]. Cells were seeded in complete medium and treated for 24 h with drug concentrations not affecting viability in that time period. Cells were transferred (8 × 10^5^ per well) to 24-well transwell chambers (Costar, Corning, Inc., Corning, NY, USA) in serum-free medium. For invasion assays, the transwell membranes were previously coated with 60 μL of 208 mg/mL matrigel per well (BD Biosciences, San Jose, CA, USA) and dried for 1 h. After 24 h of incubation at 37 °C, cells that migrated to the lower chamber or invaded the matrigel and then migrated to the lower chamber were fixed in 95% ethanol, stained with a solution of 0.4% sulforhodamine B (SRB, Sigma-Aldrich, Milan, Italy) in 0.1% acetic acid, and counted under an inverted microscope. Each experiment was carried out in triplicate, counting three fields per sample.

### 4.4. Western Blot Analyses

Western blot analysis was carried out as previously reported, with minor modifications [27]. Cells were harvested using a scraper and lyzed in a buffer composed of 0.125 M Tris HCl pH 6.8 (Sigma-Aldrich), 5% sodium dodecyl sulfate (SDS, Lonza, Verviers, Belgium) and protease/phosphatase inhibitors (Sigma-Aldrich). Protein samples were boiled for 5 min, sonicated for 25 s and quantified through the BCA assay method (Pierce, Thermo Fisher Scientific, Monza, Italy). Equal amounts of proteins were fractionated by SDS-PAGE and blotted on nitrocellulose membranes. Blots were pre-blocked in PBS containing 5% (*w*/*v*) dried nonfat milk and then incubated overnight at 4 °C with primary antibodies. Immunoreactive bands were revealed by enhanced chemiluminescence detection system ECL (GE Healthcare, Little Chalfont, UK). Antibody binding to blots was detected by chemiluminescence. The anti VEGFR2 antibody was from Bioss (Aurogene SRL, Rome, Italy), the anti-paxillin and anti-phosphopaxillin antibodies were form Abcam Limited (Cambridge, UK) and Upstate Biotechnology (Lake Placid, NY, USA), respectively. Vinculin and tubulin (Sigma-Aldrich) were used as loading control. Quantification of band intensity was performed using the ImageJ 1.47v software (http://imagej.nih.gov/ij), which attributes a numeric value to the band intensity. Each value is then normalized with respect to its loading control and the ratio between each sample and the control was calculated.

### 4.5. Analysis of Integrin Levels

The expression of integrins was measured by flow cytometry, as previously described [13]. Exponentially growing cells were harvested and incubated for 30 min at 4 °C with anti-human αvβ_3_ (Millipore Merck, Milan, Italy) or isotypic control (Sigma-Aldrich). Cells were then washed, and samples were immediately used for flow cytometric analysis (BDAccuri C6, Becton-Dickinson, Milan, Italy). Expression of integrins was expressed as the ratio between the mean fluorescence intensity obtained in cells incubated with the anti-integrin antibody divided by that of cells incubated with isotypic control.

### 4.6. Antitumor Activity Evaluation

All experiments were carried out using female athymic CD-1 nude mice, 8–10 weeks-old (Charles River, Calco, Italy). Mice were maintained in laminar flow rooms keeping temperature and humidity constant. Mice had free access to food and water. Experiments were authorized by the Italian Ministry of Health according to the national law (Project Identification: 1103/2015-PR.; approved by the Ministry of Health on 10 October 2015) in compliance with international policies and guidelines. The IGROV-1Pt1, cisplatin-resistant variant of the IGROV-1 human ovarian carcinoma, was used as xenograft in the study. Exponentially growing cells (10^7^/mouse) were s.c. injected into the right flank on day 0 forming experimental groups of seven animals. Tumor take was 100%. Tumor growth was followed by biweekly measurements of tumor diameters with a Vernier caliper. Tumor volume (TV) was calculated according to the formula: TV (mm^3^) = d^2^ × D/2 where d and D are the shortest and the longest diameter, respectively. Treatment started 4 days after cell inoculum, when tumors were not measurable. Compound **3**, 20 mg/kg i.p., and sunitinib, 40 mg/kg orally, were administered daily for 5 days a week for 4 weeks (qd × 5/w × 4w). Cisplatin (4.5 mg/Kg) was administered by i.v. three times every 7 days. The efficacy of the drug treatment was assessed as tumor volume inhibition percentage (TVI%) in treated versus control mice, calculated as: TVI% = 100−(mean TV treated/mean TV control × 100). Toxic effects of the treatment were determined as body weight loss and lethal toxicity. The highest body weight loss percentage induced by treatments is reported in Table 1. Deaths occurring in treated mice before the death of the first control mouse were ascribed to toxic effects. Student’s *t*-test (two-tailed) was used for statistical comparison of tumor volumes in mice.

### 4.7. Immunohistochemistry and Digital Image Analysis

Subcutaneous IGROV-1/Pt1 xenograft samples from the three groups were collected 24 h after the last treatment, fixed in 10% neutral buffered formalin and paraffin embedded for histopathological analyses. 4 μm sections from each sample were stained by immunohistochemistry to detect tumoral vessels with the rat anti-CD31 antibody (clone SZ31, Dianova, DIA310, dilution 1:100). For immunohistochemistry, deparaffinization, rehydration and antigen retrieval were performed in a single step method: sections were immersed for 40 min at 94 °C in a pH 9 buffer solution (Dewax and HIER Buffer H, Thermo Scientific). Endogenous peroxidase activity was blocked by incubating sections in 3% H_2_O_2_ for 15 min. To reduce nonspecific background staining, slides were rinsed and treated, for 30 min, with 10% normal rabbit serum and then incubated for 1 h at room temperature with the primary antibody. A rabbit anti-rat biotinylated secondary antibody (BA-4001, Vector Laboratories-DBA Italia, Milan, Italy) was then added for 30 min and sections were labeled by the avidin-biotin-peroxidase (ABC) procedure with a commercial immunoperoxidase kit (Vectastain Standard Elite, Vector Laboratories). The immunoreaction was developed with 3,3′-diaminobenzidine substrate (DAB, Vector Laboratories) for 5 min and sections were counterstained with Mayer’s hematoxylin. The tumor-associated vasculature was evaluated in each sample by measuring the percentage of CD31-immunolabeled area (endothelial area) in three 200× microscopic fields, randomly selected throughout the neoplastic tissue, using ImageJ software.

### 4.8. Statistical Analyses

Statistical analyses were carried out using GraphPad Prism (version 5.02, GraphPad Software Inc., La Jolla, CA, USA), as detailed throughout the manuscript.

## 5. Conclusions

Since α_V_ integrins and VEGFR2 can be overexpressed on the surface of ovarian cancer cells, here we evaluated rationally designed dual conjugates characterized by the capability to both target α_V_β_3_ integrin-expressing tumors and to inhibit VEGFR2. Our approach reflects the need to tailor treatment to the specific molecular alterations of tumors cells and to direct the drug to the tumor site, two major goals in the perspective of a personalized treatment devoid of relevant side effects. In vitro growth inhibition assays in cisplatin-sensitive and -resistant ovarian carcinoma cells showed remarkable activity associated with strong inhibition of aggressive cell features. Inhibition of tumor cell aggressiveness was not limited to cellular models expressing high levels of VEGFR2 and α_V_β_3_ integrin, since it was observed even in cell types expressing lower but still detectable levels of these receptors.

In vivo evaluation of conjugated compound **3** supported the promising features of this agent, since a marked antitumor activity was found in the cisplatin-resistant model using a dose of conjugate corresponding to a drug loading 9 times lower than the dose of sunitinib malate administered at its optimal dose. Also, significant vascular alterations induced by the treatment with compound **3** emphasized an anti-angiogenic effect in the tumor microenvironment. Due to the effective intervention of compound **3** toward drug-resistant cells by exploitation of specific tumor cell alterations, interesting perspectives may be opened, pointing to the use of dual conjugates of type **3** in the cisplatin-resistant ovarian carcinoma clinical setting, as either anti-tumor/anti-angiogenesis agents per se, or in combination with cisplatin. Further studies are necessary, however, to deeply investigate drug combination schedules and mechanisms underlying their interaction.

## Figures and Tables

**Table 1:** Effects of i.p. compound **3**, oral sunitinib and intravenous cisplatin in nude mice s.c. bearing the IGROV-1Pt1 human ovarian carcinoma.

Drug	Days of Treatment	Dose (mg/Kg)	TVI ^2^	*p*^3^ vs. Controls	BWL% ^4^	Tox ^5^
Compound **3**	4–8, 11–15, 18–22, 25–27	20	60	0.0001	1	0/7
Sunitinib	4–8, 11–15, 18–22, 25–27	40	84	0.0001	0	0/7
Cisplatin	4, 11, 18	4.5	67	0.0001	0	0/7

^1^ Tumor cells (10^7^/mouse) were s.c. inoculated into the right flank on day 0. Treatment started when tumors were not measurable. ^2^ Tumor volume inhibition % in treated over control mice assessed 20 days after cell injection. ^3^ By two-tail Student’s *t*-test. ^4^ Body weight-loss percentage induced by treatment; the highest value is reported. ^5^ Dead/treated mice.

## Supplementary Materials

The following are available online at https://www.mdpi.com/2072-6694/11/4/531/s1, Figure S1: Flow cytometric analysis of integrin α_V_β_3_ levels in IGROV-1/Pt1 and A2780 cells, Figure S2: Modulation of migratory and invasive abilities of A2780 cells by sunitinib and selective binders of α_V_β_3_ integrin linked to sunitinib, Figure S3: Whole Western blot analysis of VEGFR2 levels in different ovarian carcinoma cell lines, Figure S4: Whole Western blot analysis of paxillin and phospho-paxillin levels in IGROV-1 and IGROV-1/Pt1 cells, Table S1: Band intensities quantification of VEGFR2 levels in different ovarian carcinoma cells, Table S2: Band intensities quantification of paxillin and phosphorylated-paxillin levels in IGROV-1 and IGROV-1/Pt1 cells, Table S3: Sensitivity of the A2780 ovarian carcinoma cell line to sunitinib and conjugates **1**–**3**.
