# Supplementary material for: Efficacy of a Selective Binder of αVβ3 Integrin Linked to the Tyrosine Kinase Inhibitor Sunitinib in Ovarian Carcinoma Preclinical Models
Source: Cancers (Basel). 2019 Apr 13;11(4):531. doi: 10.3390/cancers11040531 (PMC6521192; doi:10.3390/cancers11040531)
Supplement: Supplementary file 1 [file cancers-11-00531-s001.pdf]

cancers

Supplementary Materials: Efficacy of a Selective Binder of $\alpha v\beta_3$ Integrin Linked to the Tyrosine Kinase Inhibitor Sunitinib in Ovarian Carcinoma Preclinical Models

Andrea Sartori, Cristina Corno, Michelandrea De Cesare, Eugenio Scanziani, Lucia Minoli, Lucia Battistini, Franca Zanardi and Paola Perego

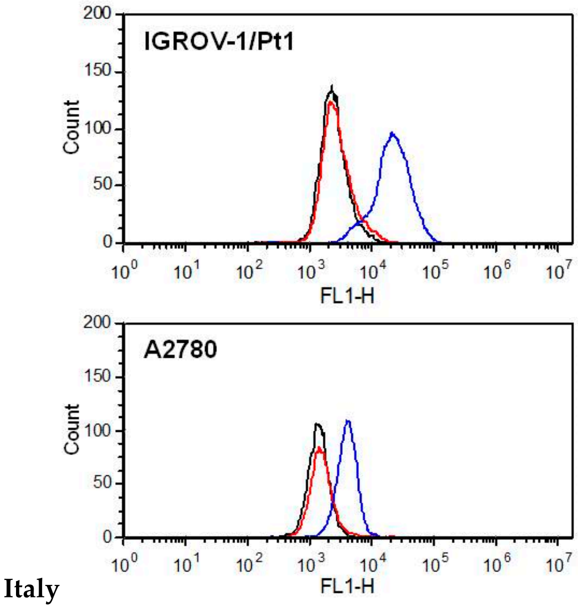

Italy

Figure S1. Flow cytometric analysis of integrin $\alpha v\beta_3$ levels in IGROV-1/Pt1 and A2780 cells. Cells were harvested and incubated for 30 min with a FITC labelled antibody (blue) or isotypic control (red). Black, cells incubated with PBS. The ratio between the mean fluorescence intensity obtained in cells incubated with the anti-integrin antibody divided by that of cells incubated with isotypic control was 14.25 and 2.4 for IGROV-1/Pt1 and A2780 cells, respectively.

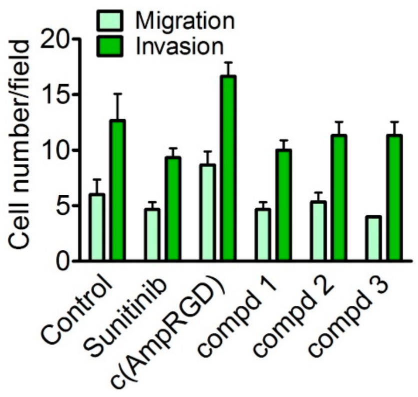

Figure S2. Modulation of migratory and invasive abilities of A2780 cells by sunitinib and selective binders of $\alpha v\beta_3$ integrin linked to sunitinib. Cells were subjected to migration (light green) and invasion assays (green) in serum-free medium using transwell plates after exposure to the compounds (0.3 μM). Migrating and invading cells were counted under a light microscope. Columns represent cell numbers/field (± SE; $n = 3$). $p < 0.05$ by Student's t test c(AmpRGD)-treated cells versus compound **1**/**2**/**3**-treated cells ($n = 6$) for both migration and invasion assays.

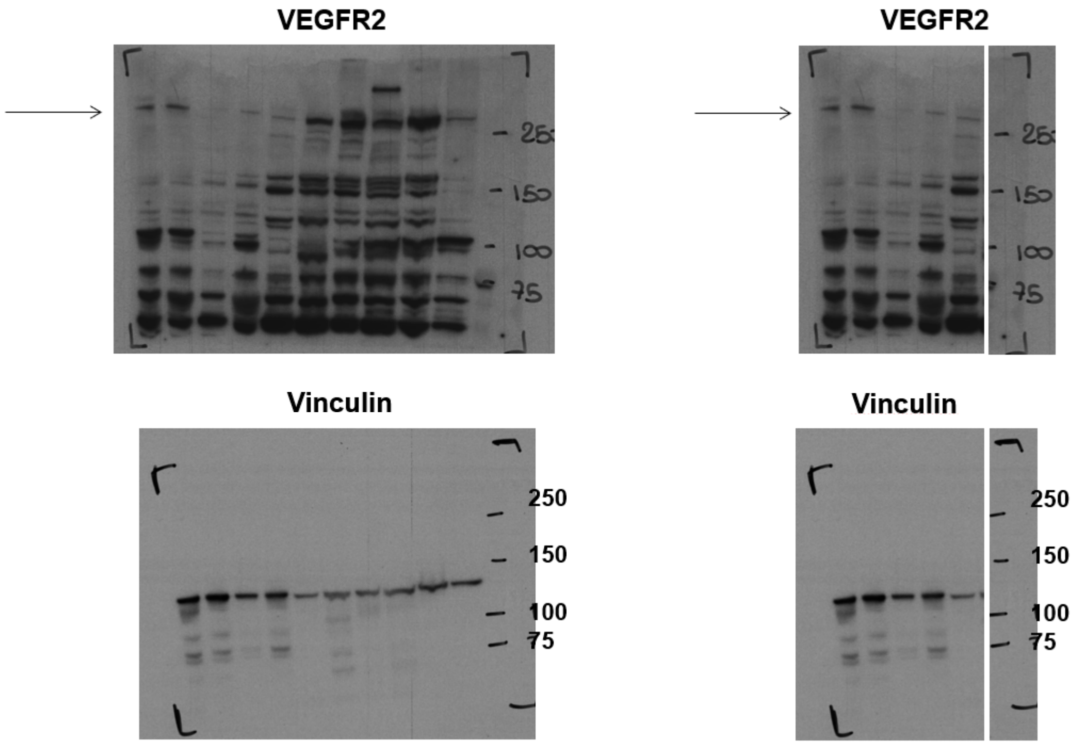

Figure S3. Whole Western blot analysis of VEGFR2 levels in different ovarian carcinoma cell lines. On the right side, the first 5 blots are selected, which are referred to the 5 cell lines reported in Figure 2 of the main text.

IGROV-1

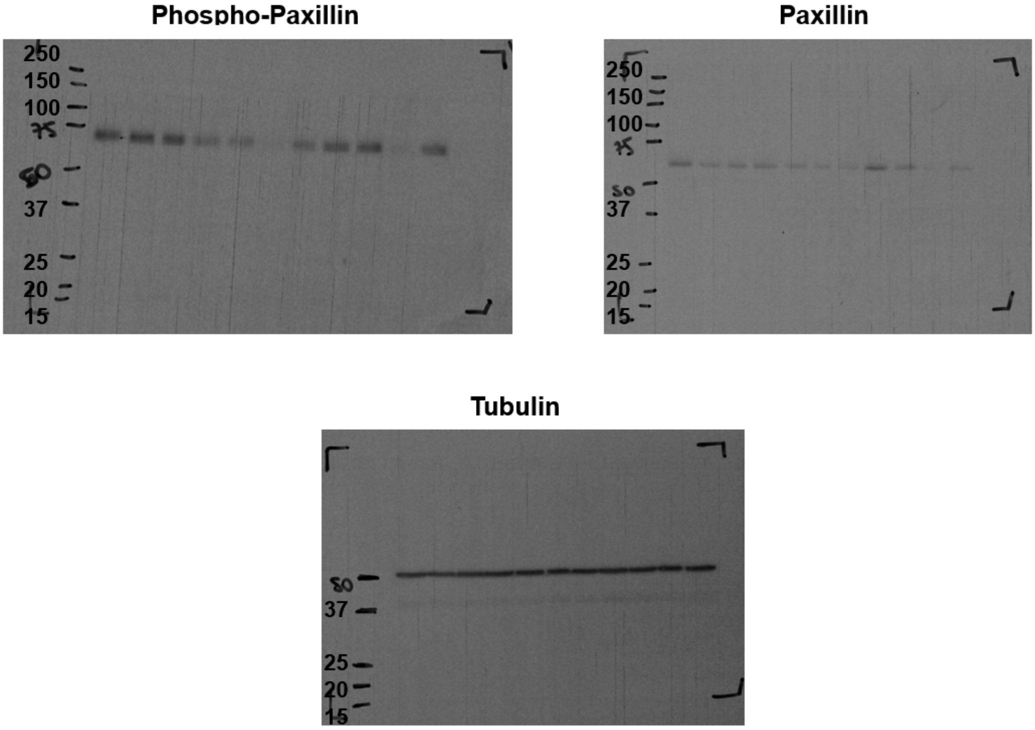

IGROV-1/Pt1

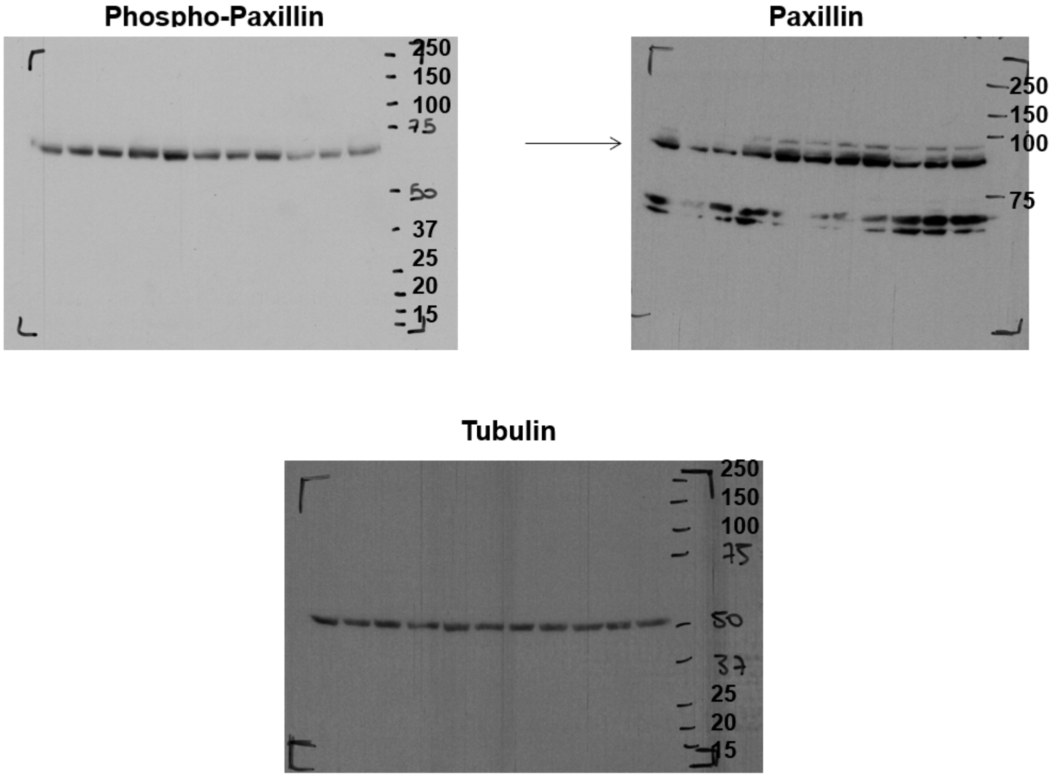

Figure S4. Whole Western blot analysis of paxillin and phospho-paxillin levels in IGROV-1 and IGROV-1/Pt1 cells (see Figure 5 in the main text).

Table S1. Band intensities quantification of VEGFR2 levels in different ovarian carcinoma cells [1].

Cell Lines	VEGFR2
**IGROV-1**	0.23
**IGROV-1/Pt1**	0.22
**A2780**	0.02
**A2780/BBR**	0.10
**A2780/CP**	0.23

[1] Western blot analysis of VEGFR2 levels in different ovarian carcinoma cells. Cells were harvested for western blot analysis. Vinculin was used as loading control. Band intensities, quantified using ImageJ, were normalized to vinculin (see Figure 2 in the main text).

Table S2. Band intensities quantification of paxillin and phosphorylated-paxillin levels in IGROV-1 and IGROV-1/Pt1 cells [1].

Compound	IGROV-1			IGROV-1/Pt1		
	p-paxillin	paxillin	p-pax/pax	p-paxillin	paxillin	p-pax/pax
**Control**	1.75	1.06	1.65	2.63	3.05	0.86
**10 μM sunitinib**	1.90	0.54	3.51	9.70	3.10	3.13
**3 μM sunitinib**	1.54	0.52	2.99	4.11	1.70	2.42
**10 μM c(Amp)RGD**	0.48	0.44	1.09	3.10	2.36	1.31
**3 μM c(Amp)RGD**	0.45	0.21	2.12	6.58	6.18	1.06
**10 μM compd1**	0.05	0.12	0.39	3.29	4.93	0.67
**3 μM compd1**	0.58	0.14	4.08	3.50	6.71	0.52
**10 μM compd2**	1.12	0.80	1.40	1.53	2.82	0.54
**3 μM compd2**	1.23	0.36	3.45	0.54	1.72	0.31
**10 μM compd3**	0.068	0.058	1.17	0.73	1.78	0.41
**3 μM compd3**	1.06	0.22	4.82	0.57	1.09	0.52

[1] Western blot analysis of paxillin and phosphorylated-paxillin (p-paxillin) levels in IGROV-1 and IGROV-1/Pt1 cells. Cells were exposed for 24 h to sunitinib or to selective binders of $\alpha_v\beta_3$ integrin linked to sunitinib (compounds **1**–**3**) at the indicated concentrations and then harvested for western blot analysis. Tubulin was used as loading control. Band intensities, quantified using ImageJ, were normalized to tubulin (see Figure 5 in the main text).

Table S3. Sensitivity of the A2780 ovarian carcinoma cell line to sunitinib and conjugates **1–3** [1].

IC_{50} (μM) [1]			
**Sunitinib**	**Compound 1**	**Compound 2**	**Compound 3**
0.62 ± 0.02	0.65 ± 0.2	2.22 ± 1.1	1.33 ± 0.17

[1] Sensitivity to sunitinib or the conjugates was assessed by cell growth inhibition assays. Cells were seeded and 24 h later exposed to the compounds for 72 h. The IC_{50} value of c(AmpRGD) *per se* was 3.48 ± 0.25 μM. Cells were then counted using a cell counter. IC_{50} is defined as the concentration inhibiting cell growth by 50%.

© 2019 by the authors. Licensee MDPI, Basel, Switzerland. This article is an open access article distributed under the terms and conditions of the Creative Commons Attribution (CC BY) license (http://creativecommons.org/licenses/by/4.0/).
